# Effects of chondroitin sulfate oligosaccharides on osteoclast differentiation of RAW264 cells, and myotube differentiation of C2C12 cells

**DOI:** 10.1371/journal.pone.0284343

**Published:** 2023-04-13

**Authors:** Hirofumi Uchiyama, Daisuke Muramatsu, Hideaki Higashi, Hiroshi Kida, Atsushi Iwai

**Affiliations:** 1 Aureo Science Co., Ltd., Kita-ku, Sapporo, Hokkaido, Japan; 2 Division of Bioscience in Sapporo, Aureo Co., Ltd., Kita-ku, Sapporo, Hokkaido, Japan; 3 Division of Infection and Immunity, International Institute for Zoonosis Control, Hokkaido University, Kita-ku, Sapporo, Hokkaido, Japan; 4 International Institute for Zoonosis Control, Hokkaido University, Kita-ku, Sapporo, Hokkaido, Japan; Northwest University, UNITED STATES

## Abstract

Chondroitin sulfate (CS) is a glycosaminoglycan, and CS derived from various animal species is used in drugs and food supplements to alleviate arthralgia. The CS is a high molecular weight compound, and hydrolysis of CS by intestinal microbiota is thought to be required for absorption in mammalians. Chondroitin sulfate oligosaccharides (Oligo-CS) are produced by hydrolysis with subcritical water from CS isolated from a species of skate, *Raja pulchra* for the improvement of bioavailability. The present study conducted in vitro experiments using murine cell lines, to compare the biological activities of Oligo-CS and high molecular weight CS composed with the similar disaccharide isomer units of D-glucuronic acid and N-acetyl-D-glucosamine (CS-C). The results show that Oligo-CS inhibits osteoclast differentiation of RAW264 cells significantly at lower concentrations than in CS. The cell viability of a myoblast cell line, C2C12 cells, was increased when the cells were grown in a differentiated medium for myotubes with Oligo-CS, where there were no effects on the cell viability in CS. These results suggest that in vitro Oligo-CS exhibits stronger bioactivity than high-molecular weight CS.

## Introduction

**Fig 1:**
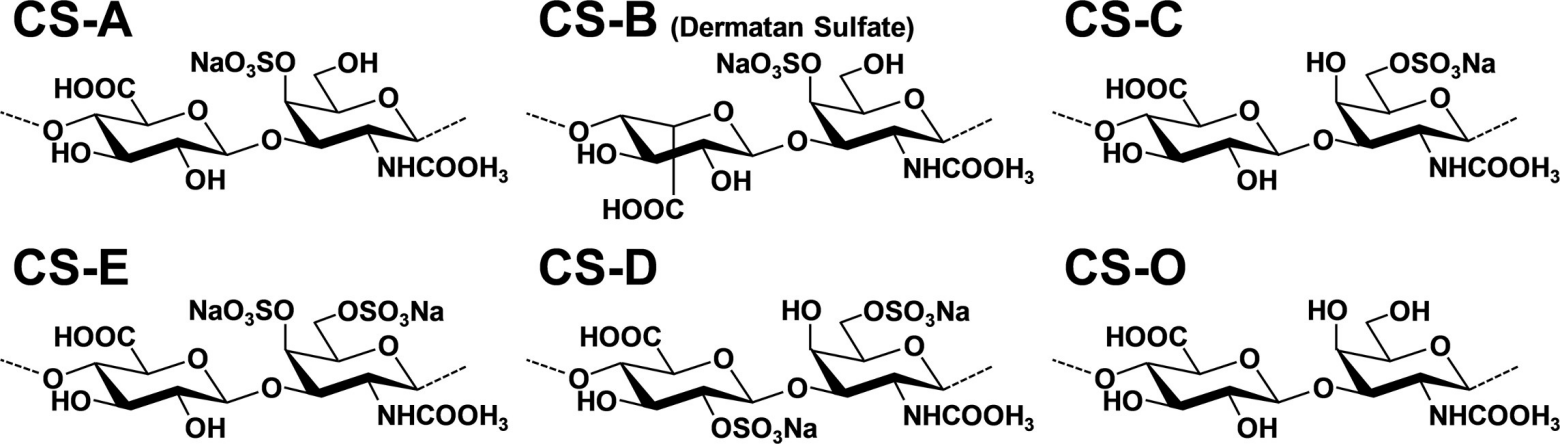
Molecular structure of CS (sodium salt) structural isomer variants.

Chondroitin sulfate (CS) is a glycosaminoglycan, and it is commonly present as a proteoglycan. The CS proteoglycans are abundantly present in cartilage as an extracellular matrix, and are important components of the cartilage that maintains joint structure. The CS consists of repeating disaccharide units (D-glucuronic acid and N-acetyl-D-glucosamine) with sulfate residues, and there are several disaccharide unit isomers in CS depending on its ester-linked sulfate positions (Fig 1) [1–3]. The structure of repeating disaccharide units in CS is different depending on its origin [4, 5].

The CS extracted from the cartilage of various animal species such as bovine, porcine, chicken, shark, and skate is used in drugs and food supplements to alleviate arthralgia [6, 7]. Cartilaginous fish including sharks, rays, and skates are thought to be a good natural source of CS [8]. The chondroitin sulfate oligosaccharides (Oligo-CS) used in this study were produced by hydrolysis with subcritical water from CS isolated from a skate species, *Raja pulchra* [9]. Oligo-CS is thought to be more effectively absorbed in the small intestine than high molecular weight CS, and the effects of oral administration of Oligo-CS on improvements in knee pain and locomotive syndrome cases at low doses have been demonstrated by a human intervention study [10]. In this present study, to assess the biological activities of Oligo-CS compared to high molecular weight CS, the inhibitory activity on the differentiation of RAW264 cells into osteoclasts, and the effects on the differentiation of C2C12 cells into myotubes were investigated.

## Materials and methods

### Chondroitin sulfate (CS) and chondroitin sulfate oligosaccharides (Oligo-CS)

The Oligo-CS, chondroitin sulfate oligosaccharides (Marukyou Bio Foods, Wakkanai, Hokkaido, Japan) and CS, chondroitin sulfate C sodium salt (Fujifilm Wako pure chemical corporation, Osaka, Japan) used in this study are commercially available products.

### Cell lines

The RAW264 cells (a mouse leukemic monocyte cell line; ECA85062803) [11] were purchased from RIKEN BRC through the National BioResource Project of the MEXT, Japan. The RAW264 cells were maintained in RPMI1640 medium supplemented with 10% fetal bovine serum (FBS; Biowest, Nuaillé, France), 100 U/ml penicillin, and 100 mg/ml streptomycin (Life Technologies, Carlsbad, CA, USA). The immortalized mouse myoblast cell line, C2C12 cells (ATCC CRL-1772) [12], was obtained from the JCRB (Japanese collection of research bioresources) cell bank (Tokyo, Japan). The C2C12 cells were maintained in D-MEM medium supplemented with 10% FBS, 100 U/ml penicillin, and 100 mg/ml streptomycin. These cells were grown at 37°C in 5% CO_2_ in a humidified incubator.

### Tartrate-resistant acid phosphatase (TRAP) staining

To differentiate the osteoclasts, RAW264 cells were seeded on 24-well plates in phenol red-free α-MEM supplemented with 10% FBS. After overnight incubation, the cells were stimulated with 2.5 nM soluble RANKL (sRANKL, PeproTech, Rocky Hill, NJ, USA) and test samples. At 5 days after the stimulation, the cells were subjected to TRAP staining. The TRAP staining was performed using a commercially available kit (TRAP Staining Kit; Cosmo bio, Tokyo, Japan) following the manufacturer protocol. In this study, TRAP positive, multinuclear and large cytoplasm cells were defined as mature osteoclasts and others as immature osteoclasts.

### Real time RT-PCR analysis

Total RNA was isolated from the cells using Trizol reagent (Thermo Fisher Scientific, Waltham, MA, USA). The isolated total RNA was treated with DNaseI (Takara, Otsu, Shiga, Japan), and then subjected to an oligo-dT and random primed reverse transcriptase reaction using ReverTra Ace (Toyobo, Osaka, Japan). Real-time PCR was performed on the CFX96 Real-Time PCR Detection System (Bio-Rad, Hercules, CA, USA) using Thunderbird SYBR qPCR Mix (Toyobo). These procedures were performed according to the manufacturer instructions. Specific primer sets were used in this study as listed in Table 1.

**Table 1:** Specific primer sets used in this study.

Target Gene		Sequence (5’ → 3’)
***TRAP***	**Sense**	**GCGACCATTGTTAGCCACATACG**
	**Antisense**	**CGTTGATGTCGCACAGAGGGAT**
***CTSK***	**Sense**	**GGGCCAGGATGAAAGTTGTA**
	**Antisense**	**CCGAGCCAAGAGAGCATATC**
***MMP-9***	**Sense**	**GCTGACTACGATAAGGACGGCA**
	**Antisense**	**GCGGCCCTCAAAGATGAACGG**
***MyoD***	**Sense**	**AACTGCTCTGATGGCATGATG**
	**Antisense**	**TGGAGATGCGCTCCACTATG**
***Myf5***	**Sense**	**CTCTGAAGGATGGACATGACGG**
	**Antisense**	**ACTGGTCCCCAAACTCATCCTC**
***Msx2***	**Sense**	**TCACCACGTCCCAGCTTCTAG**
	**Antisense**	**AGCTTTTCCAGTTCCGCCTCC**
***Sox9***	**Sense**	**ATCTGAAGAAGGAGAGCGAG**
	**Antisense**	**TCAGAAGTCTCCAGAGCTTG**
***Runx2***	**Sense**	**GCGTCAACACCATCATTCTG**
	**Antisense**	**CAGACCAGCAGCACTCCAT**
***PPARγ***	**Sense**	**GCCCTTTGGTGACTTTATGGA**
	**Antisense**	**GCAGCAGGTTGTCTTGGATG**

### Western blotting analysis and monitoring of cell viability

To differentiate the C2C12 cells to myotube, cells were grown in D-MEM medium supplemented with 2% FBS, 1 nM insulin. After 72 hours, the cells were harvested, and lysed with radioimmune precipitation (RIPA) buffer (25 mM Tris-HCl [pH 7.5], 150 mM NaCl, 1% Nonidet P-40, 1% sodium deoxycholate, 0.1% sodium dodecyl sulfate [SDS]) supplemented with a cOmplete Mini protease inhibitor cocktail tablet (Sigma-Aldrich; Merck KGaA, Darmstadt, Germany). After centrifugation to remove debris, the protein concentration of the samples was determined using a BCA protein assay kit (Thermo Fisher Scientific), and a 10 μg protein/sample was subjected to Western blotting analysis. Mouse monoclonal antibodies, anti-human myosin heavy chain antibody (sc-376157; Santa Cruz Biotechnology, Santa Cruz, CA, USA), and anti-β-actin antibody (A5316; Sigma-Aldrich) were used for the detection of MYH and β-actin proteins respectively. Anti-MYH antibody was used at 1:1000 dilution, and anti-β-actin antibody was used at 1:5000 dilution. Horseradish peroxidase (HRP) conjugated Goat polyclonal antibody against mouse IgG (HAF007; R&D Systems, Minneapolis, MN, USA) was used as a secondary antibody at 1:10000 dilution. Restore Western Blot Stripping Buffer (Thermo Fisher Scientific) was used for reprobing to detect β-actin after the MYH detection.

The cell viability was monitored using a Cell Counting Kit-8 (Dojindo, Kumamoto, Japan) in accordance with the manufacturer instructions.

### Oil Red O staining

For the differentiation of C2C12 cells into adipocytes, the cells were grown in D-MEM supplemented with 10% FBS, 0.5 mM 3-isobutyl-1-methylxanthine (IBMX), 1 μM dexamethasone, and 10 μg/mL insulin for 2 days. Then the cells were grown in D-MEM supplemented with 10% FBS, and 10 μg/mL insulin for an additional 3 days. The cells were washed twice with phosphate buffered saline (PBS), and fixed with 10% formaldehyde for 10 min at room temperature. After the cells were washed three times with distilled water, the cells were stained with Oil Red O for 15 min. The cells were washed four times with distilled water, then the stained Oil Red O was extracted from the cells with isopropanol, and lipid accumulation in the cells was estimated by measuring absorbances at 540 nm using a multiplate reader.

### Statistical analysis

In this study, the unpaired two-tailed Student’s t test with Welch’s correction was used to determine whether differences were statistically significant. A *p*-value smaller than 0.05 was considered to show statistically significant differences.

## Results

### Oligo-CS effectively inhibits osteoclast differentiation of RAW264 cells

Receptor activator of nuclear factor-κB (RANK) and RANK ligand (RANKL) mediated signaling pathway is essential for terminal differentiation and maturation of osteoclast through the induction of the RANKL-induced nuclear factor of activated T cells, cytoplasmic 1 (NFATc1) [13]. The NFATc1 is known as a master transcription regulator of osteoclast differentiation, and involved in the expression of tartrate-resistant acid phosphatase (TRAP), a typical marker protein for osteoclasts, as well as cathepsin K (CTSK), and matrix metalloproteinase-9 (MMP-9) [14, 15]. The TRAP, CTSK, and MMP-9 proteins are abundantly expressed in mature osteoclasts, and involved in the osteoclast functions of the bone resorption.

**Fig 2:**
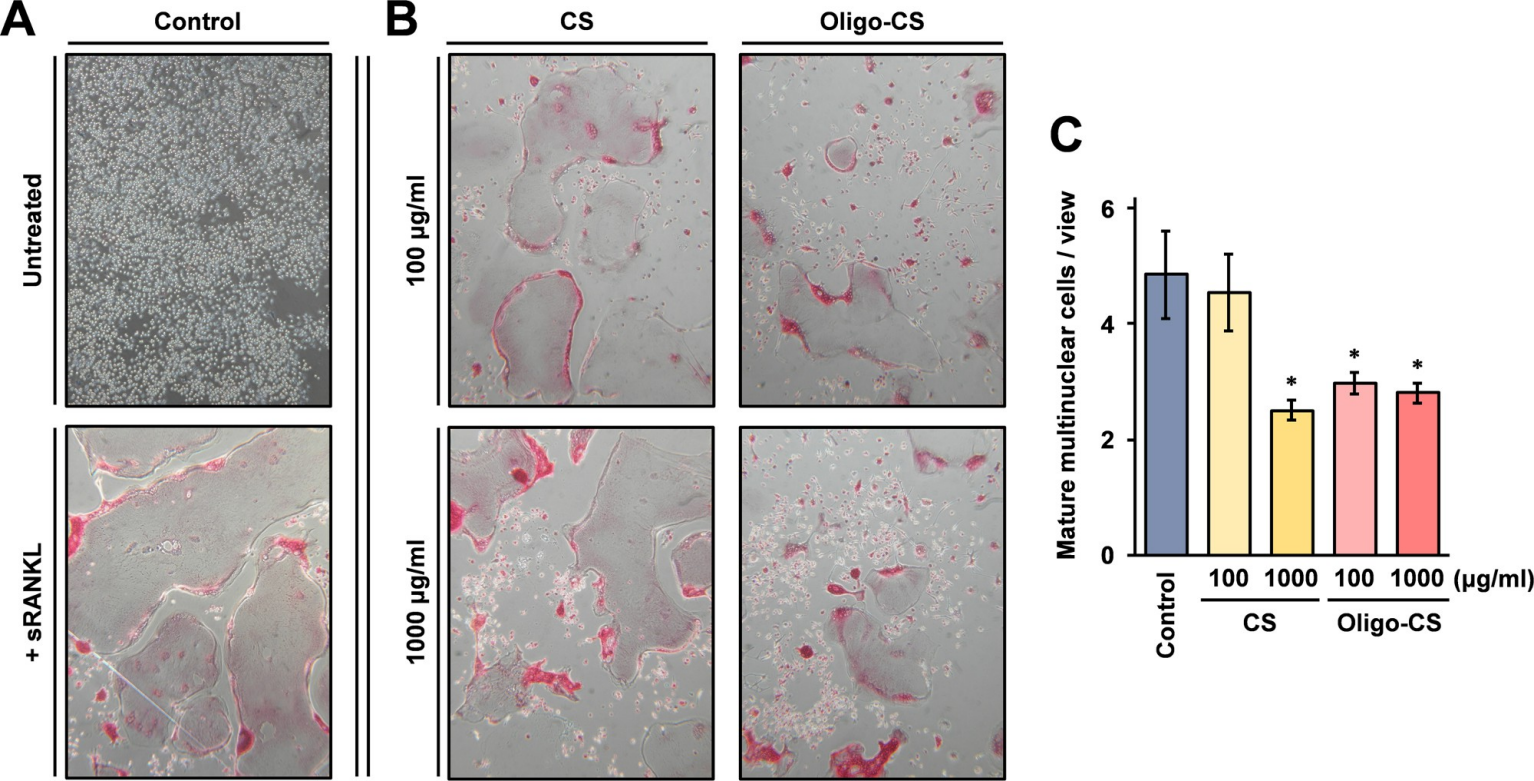
Oligo-CS effectively inhibits differentiation of RAW264 cells into osteoclasts. (A) RAW264 cells were grown in differentiation medium for 5 days to differentiate into osteoclasts. The control cells were grown in differentiation medium without sRANKL. Then the cells were subjected to TRAP staining. (B) RAW264 cells were grown in differentiation medium with the indicated concentrations of CS or Oligo-CS for 5 days, and the cells were subjected to TRAP staining. (C) The TRAP positive mature multinuclear cells were counted under a microscope. Error bars indicate standard deviations calculated from at least 19 frames of each group. Asterisks (*) shown in the graph indicate that the difference is statistically significant (*p* < 0.05) compared to the control.

A murine macrophage-like cell line, RAW264 cells express RANK at high levels, and are differentiated into osteoclasts after stimulation with RANKL [16]. As shown in Fig 2A, RAW264 cells are differentiated to osteoclasts in the presence of soluble RANKL. Oligo-CS is mainly composed of CS-C, and the formulations are reported as CS-C (71.9%), CS-O (10.8%), CS-D (8.8%), CS-A (8.5%) [5]. A previous report demonstrated weak inhibitory activity of CS-C on osteoclast differentiation of RAW264 cells [17]. To assess the effects of oligomerization of CS-C, inhibition of Oligo-CS on osteoclast differentiation of RAW264 cells was investigated. The RAW264 cells were differentiated into osteoclasts when growing in the differentiation medium in the presence of Oligo-CS, and the osteoclast differentiation was evaluated by tartrate-resistant acid phosphatase (TRAP) staining and microscopy. Microscopy images are shown in Fig 2B, and the results show that the numbers (frequency of appearance) of small cells which are thought to be immature osteoclasts are increased by the CS and Oligo-CS treatment. As shown in Fig 2C, the image analysis results demonstrated that both high molecular weight CS and Oligo-CS were significantly inhibited in the osteoclast differentiation at a high concentration (1,000 μg/ml), whereas only Oligo-CS inhibited the osteoclast differentiation at the low concentration (100 μg/ml). The results indicate that treatment with Oligo-CS more effectively inhibits osteoclast differentiation of RAW264 cells more than with CS treatment.

### Oligo-CS treatment inhibits mRNA expression of TRAP, CTSK, and MMP-9 in osteoclast differentiated RAW264 cells

**Fig 3:**
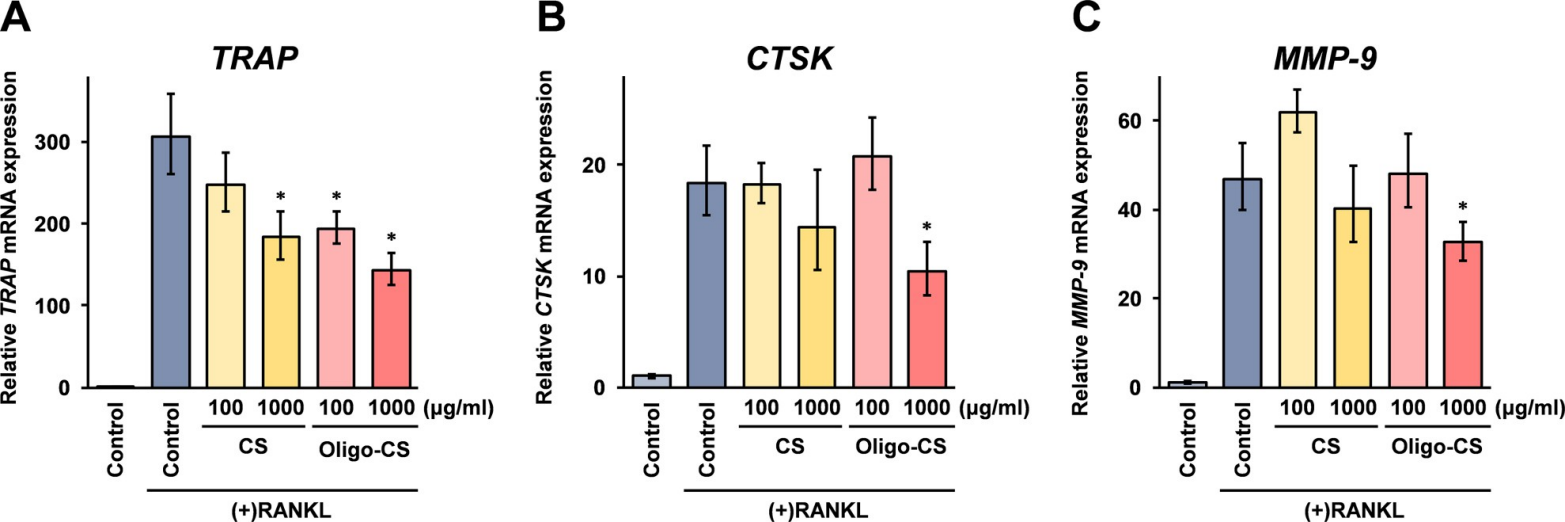
Effects of Oligo-CS treatment on TRAP, CTSK, and MMP-1 mRNA expression in osteoclast differentiating RAW264 cells. RAW264 cells were stimulated with sRANKL to differentiate osteoclasts together with indicated concentrations of CS or Oligo-CS for 5 days. The total RNA isolated from the cells was subjected to real-time RT-PCR analysis using the respective specific primer set for TRAP, CTSK, and MMP-9 mRNA. The data indicate the relative expressions compared with untreated control cells without sRANKL stimulation after normalization with the GAPDH mRNA expression. Error bars indicate the standard deviations (n = 3). Asterisks (*: *p* < 0.05) indicate that the difference is statistically significantly lower than the sRANKL stimulated control.

For further investigation of the effects of Oligo-CS on the inhibition of osteoclast differentiation of RAW264 cells, real-time RT-PCR analysis on the mRNA expression of TRAP, CTSK, and MMP-9 was performed. As shown in Fig 3A, the TRAP mRNA expression in the RAW264 cells grown in the osteoclast differentiation medium was significantly lower after the treatment with low a concentration (100 μg/ml) of Oligo-CS, as well as after treatment with a high concentration (1,000 μg/ml) of CS and Oligo-CS. These results correlated with the effects of CS and Oligo-CS on the RAW264 cell differentiation into osteoclasts after stimulation with sRANKL shown in Fig 2. However, mRNA expressions of the proteases, CTSK and MMP-1 in osteoclast differentiated RAW264 cells were significantly lower only in high concentrations of Oligo-CS treated cells (Fig 2B and 2C). These results suggest that the inhibition activity of Oligo-CS on osteoclast differentiation of RAW264 cells is stronger than with high molecular weight CS.

### Treatment with Oligo-CS in differentiation medium increases cell viability of C2C12 cells to myotubes

**Fig 4:**
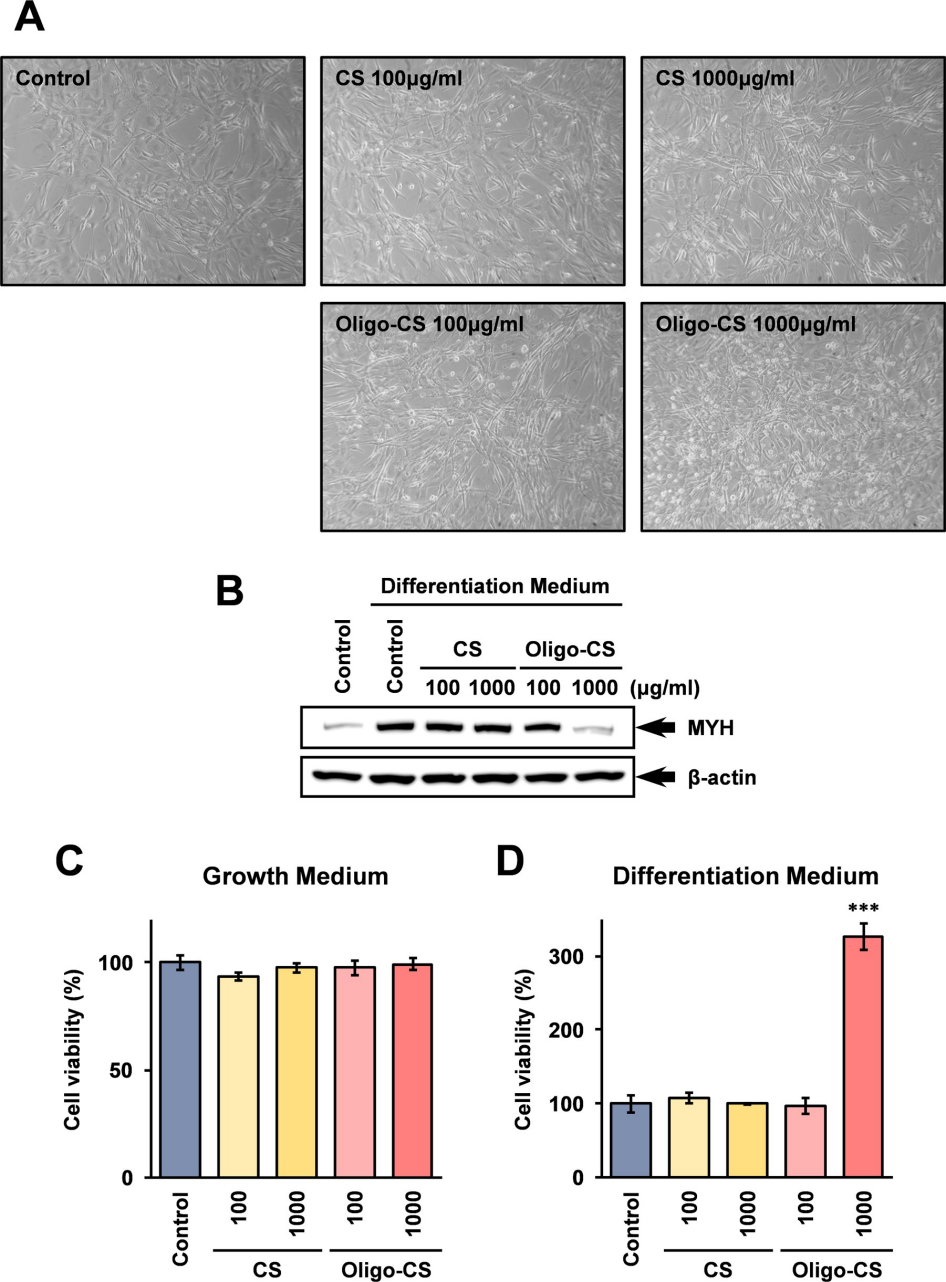
Oligo-CS increases cell viability of C2C12 cells in myotube differentiation medium. C2C12 cells were grown in the differentiation medium with the indicated concentrations of CS or Oligo-CS to differentiate into myotube cells for 72 hours as described in the Materials and Methods. (A) Morphological changes of the C2C12 cells were monitored by phase contrast microscopy. (B) Protein expression of myosin heavy chain (MYH) in C2C12 cells grown in differentiation medium was analyzed by Western blotting. The whole cell lysate containing 10 μg protein was applied to each lane. Anti-β-actin antibody was used for the loading control. (C, D) C2C12 cells were grown in growth medium (C) or differentiation medium (D) with the indicated concentration of CS or Oligo-CS for 72 hours. The cell viability of the cells was monitored using Cell Counting Kit 8. The data are shown as percentage of cell viabilities against untreated control cells. Error bars indicate standard deviations (n = 3). The triple asterisk (***) shown in the graph indicates that the difference is statistically significant (*p* < 0.001) compared to the control.

An immortalized mouse myoblast cell line, C2C12 cells are known to be differentiated to myotubes when the cells are exposed in low serum conditions [18]. In this study, C2C12 cells were grown and maintained in growth medium (D-MEM supplemented with 10% FCS), and D-MEM supplemented with 2% FCS and 1 nM insulin were used as the differentiation medium for the myotube differentiation [19]. To investigate the effects of CS and Oligo-CS on the differentiation of C2C12 cells to myotubes, C2C12 cells were grown in a differentiation medium containing CS or Oligo-CS. As shown in Fig 4A, the number of undifferentiated C2C12 cells characterized by mononuclear spherical shapes was increased in the cells grown in the differentiation medium containing 1,000 μg/ml Oligo-CS in comparison with that of the control, by observations using a phase difference microscope. To confirm these microscopy results, protein expression of myosin heavy chain (MYH) in the cells was monitored by Western blotting analysis. The results show that MYH expression was much lower in C2C12 cells grown in the differentiation medium containing 1,000 μg/ml Oligo-CS, suggesting that the population of undifferentiated myoblast-like C2C12 cells were higher in the cells treated with 1,000 μg/ml Oligo-CS (Fig 4B).

Next, the effects of CS and Oligo-CS on the cell viability of C2C12 cells in growth medium and differentiation medium were investigated using the Cell Counting Kit-8. The results show that neither of CS and Oligo-CS influenced the cell viability of C2C12 cells in the growth medium (Fig 4C). However, when C2C12 cells were grown in the differentiation medium, the viability of C2C12 cells was significantly improved in the medium containing 1,000 μg/ml Oligo-CS (Fig 4D).

### Effects of CS and Oligo-CS on the expression of marker genes in C2C12 cells grown in the differentiation medium

**Fig 5:**
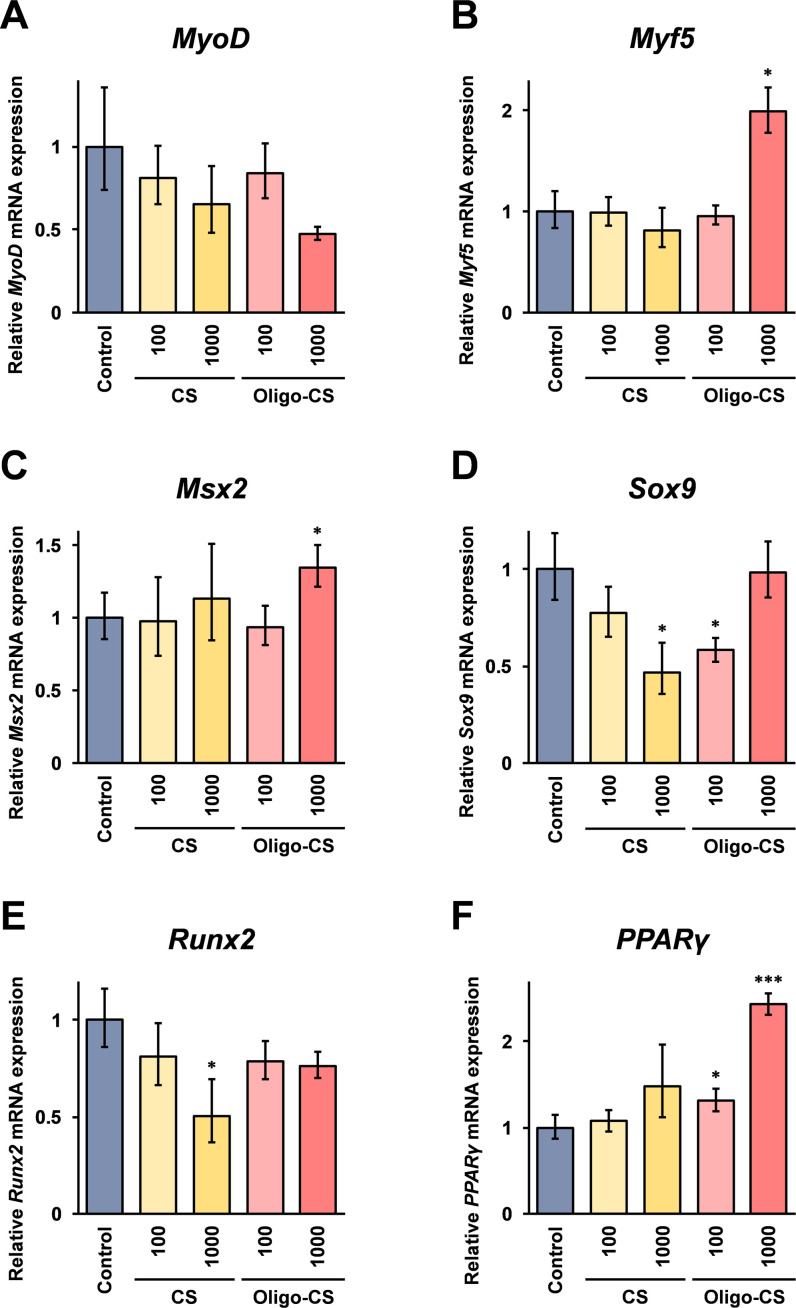
The effects of CS and Oligo-CS on mRNA expression of marker genes. C2C12 cells were grown in differentiation medium containing the indicated concentrations of CS or Oligo-CS for 72 hours. Then, the cells were harvested, and the total RNA isolated from the cells was subjected to real-time RT-PCR analysis using the specific primer set for each of the genes. The data indicate relative expressions compared to untreated control cells after normalization with the GAPDH mRNA expression. Error bars indicate standard deviations (n = 3). Asterisks (*: *p* < 0.05) and triple asterisks (***: *p* < 0.001) indicate that the difference is statistically significant compared to the control.

To understand the mechanism of the effects of Oligo-CS leading to the increase in the cell viability of C2C12 cells in the differentiation medium, expression of several marker genes involved in cell differentiation was investigated using real-time RT-PCR. Myogenic Differentiation 1 (*MyoD*) and Myogenic factor 5 (*Myof5*) are known to be necessary for myoblast differentiation [20, 21]. The real-time RT-PCR analysis results show that although the mean value of the *MyoD* mRNA expression in 1,000 μg/ml Oligo-CS treated cells decreased compared to that in control cells, the difference was not statistically significant (Fig 5A). The *Myf5* mRNA expression, however, increased significantly in the cells grown in the differentiation medium containing 1,000 μg/ml Oligo-CS (Fig 5B). Muscle segment homeobox 2 (*Msx2*) and SRY-box transcription factor 9 (*SOX9*) genes are involved in chondrocyte maturation [22, 23]. The mRNA expression of *Msx2* was weakly but significantly increased when the cells were grown in the 1000 μg/ml Oligo-CS containing differentiation medium (Fig 5C), and the *Sox9* mRNA expression was significantly decreased in 1,000 μg/ml CS treated cells, but not significantly changed in 1,000 μg/ml Oligo-CS treated cells (Fig 5D). Runt-related transcription factor 2 (*Runx2*) is associated with osteoblast differentiation [22, 23], and *Runx2* mRNA expression was significantly lower when the cells were grown in the 1,000 μg/ml CS containing medium, but not in the 1,000 μg/ml Oligo-CS containing medium (Fig 5E). The mRNA expression of peroxisome proliferator-activated receptor (γ*PPARγ*) which is known to be crucial for differentiation of adipocytes [24], was significantly increased in cells grown in the 100 and 1,000 μg/ml Oligo-CS containing medium (Fig 5F).

### The mRNA expression of marker genes in C2C12 cells differentiated to adipocytes

**Fig 6:**
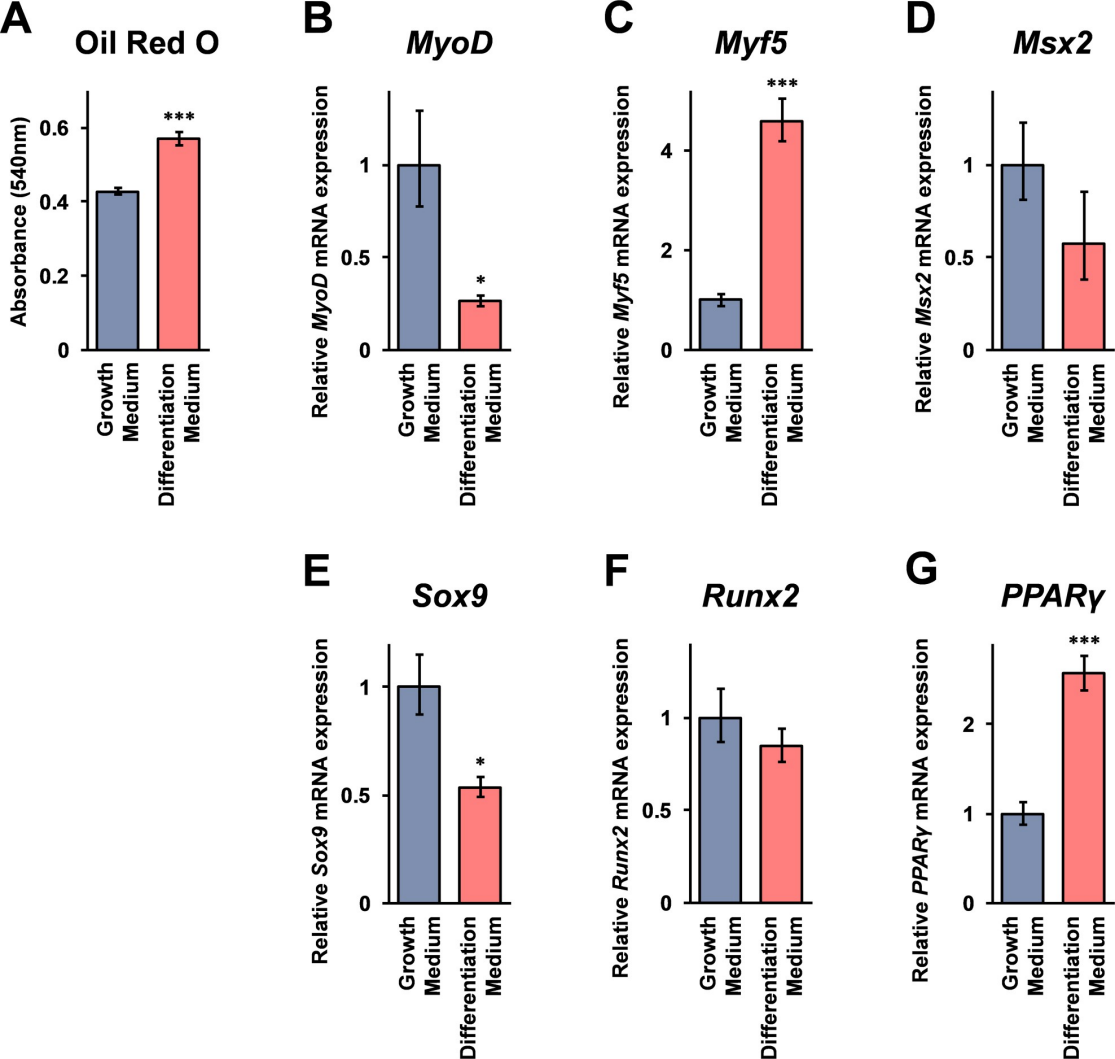
Marker gene mRNA expressions in C2C12 cells grown in adipocyte differentiation medium. (A) C2C12 cells were grown in adipocyte differentiation medium for a total of 5 days as described in the Materials and Methods. The concentrations of accumulated lipids were measured by the Oil Red O staining method. (B-G) Marker gene mRNA expressions in the C2C12 cells grown in adipocyte differentiation medium were monitored by real-time RT-PCR analysis using the specific primer set for each of the genes. The data indicate relative expressions compared to cells grown in growth medium after normalization with GAPDH mRNA expression. Error bars indicate standard deviations (n = 3). Asterisks (*: *p* < 0.05), and triple asterisks (***: *p* < 0.001) indicate that the difference is statistically significant between two groups.

As shown in Fig 5F, mRNA expression of *PPARγ*, the gene crucial for adipocyte differentiation, was significantly higher in C2C12 cells grown in the myotube differentiation medium containing Oligo-CS. This suggests the possibility that Oligo-CS has adipocyte differentiation effects on C2C12 cells. To assess this further, C2C12 cells were differentiated to adipocytes, and the mRNA expression of the marker genes indicated in Fig 5 was investigated. The results of Oil Red O staining indicate that although lipid accumulation of C2C12 cells was weak in the experimental condition (grown in the medium containing 0.5 mM 3-isobutyl-1-methylxanthine [IBMX], 1 μM dexamethasone, and 10 μg/mL insulin for 2 days, and the medium containing 10 μg/mL insulin for 3 days) used in this study, the amount of Oil Red O stained lipids was significantly increased in the C2C12 cells differentiated to adipocytes (Fig 6A). Real time RT-PCR analysis results indicate that myoblast differentiation related genes, *MyoD* mRNA expression was significantly lower (Fig 6B), and *Myf5* mRNA expression was significantly higher (Fig 6C) in the adipocytes differentiated C2C12 cells. The genes involved in chondrocyte maturation, *Msx2* mRNA expression were not significantly changed (Fig 6D), and the *Sox2* mRNA expression was significantly lower (Fig 6E) in the C2C12 cells differentiated to adipocytes, compared to undifferentiated C2C12 cells. The gene involved in osteoblast differentiation, *Runx2* mRNA expression was not significantly changed in either kind of cell (Fig 6F), and the mRNA expression of *PPARγ*, essential for adipocyte differentiation, was significantly higher in adipocyte differentiated C2C12 cells (Fig 6G).

## Discussion

The present study demonstrates that Oligo-CS, oligomerized CS derived from *Raja pulchra* is more effectively inhibiting osteoclast differentiation of RAW264 cells than high molecular weight CS-C, and that Oligo-CS increases the viability of C2C12 cells during differentiation to myotubes.

The results shown in this study demonstrate that 100 μg/ml Oligo-CS exhibits similar inhibitory activity as 1,000 μg/ml high molecular weight CS on the differentiation of RAW264 cells into osteoclasts (Fig 2C). The molecular weights of Oligo-CS and CS are different and the mole concentration may be a cause of the results. These results could be important to demonstrate that oligomerized CS exhibits similar effects as high molecular weight CS on the inhibition of osteoclast differentiation. The result shown in Fig 2C also indicates that the inhibition activity on the RAW264 cell differentiation into osteoclasts is not significantly different for 100 μg/ml and 1,000 μg/ml Oligo-CS. This result may suggest that the inhibitory activity of Oligo-CS has reached a plateau at a concentration lower than 100 μg/ml. The inhibitory activity in the differentiation of RAW264 cells to osteoclasts is different for different compositions of the disaccharide unit isomers in CS. A previous report demonstrated that CS isolated from squid cartilage mainly consisting of CS-E effectively inhibited osteoclast differentiation of RAW264 cells, while no significant inhibitory activity was detected in treatment with shark cartilage derived CS mainly composed of CS-C [17]. These findings suggest that the physiological activity is different in these CS isomers, and that CS oligomer which has a different composition of the disaccharide unit isomers from that of the Oligo-CS used in this study may exhibit different inhibitory activity on osteoclast differentiation.

As shown in Fig 3C, when C2C12 cells were grown in the myotube differentiation medium containing Oligo-CS, the cell viability of C2C12 cells was significantly higher than that in the control differentiation medium. The results of phase contrast microscopy shown in Fig 4A indicate that undifferentiated myoblast-like cells were more common in C2C12 cells grown in the differentiation medium containing Oligo-CS. These results suggest that Oligo-CS promotes myoblast proliferation and inhibits differentiation to myotubes when the cells were grown in the myotube differentiation medium. There is no inhibitory activity of high molecular weight CS on the differentiation of C2C12 cells into myotubes at the experimental conditions in this study (Fig 4). However, such inhibitory activity of CS has been reported by other researchers [25]. This would suggest that Oligo-CS also exhibits strong biological activity on the inhibition of myotube differentiation of C2C12 cells, stronger than that of high molecular weight CS.

The results of the mRNA expression analysis using real-time RT-PCR indicate that *Myf5* mRNA expression is significantly increased in the C2C12 cells grown in the myotube differentiation medium containing Oligo-CS (Fig 5B). Here it is noteworthy that *MyoD* and *Myf5* genes are known to be essential for myoblast differentiation, and the mouse lack of both genes is resulting in a complete absence of skeletal muscle [26]. A basic helix loop helix transcription factor, *Myf5* is involved in muscle regeneration through transient expression in the activation of myoblast proliferation [27]. This may suggest that the induction of *Myf5* mRNA expression is involved in the effects of Oligo-CS on the increment of cell viability of C2C12 cells grown in the myotube differentiation medium. Further, *Myf5* is also involved in the cell differentiation into adipocytes [28], and the results of the mRNA expression of the several marker genes involved in cell differentiation indicate that except the *Sox9* mRNA expression, the mRNA expression in the Oligo-CS treated C2C12 cells grown in myotube differentiation medium resembled the mRNA expressions in the C2C12 cells grown in adipocyte differentiation medium (Figs 5 and 6). A previous report demonstrated that Oligo-CS inhibits lipid accumulation in a murine pre-adipocyte cell line, 3T3-L1 cells, when the cells are grown in adipocyte differentiation medium [5]. That report demonstrates that Oligo-CS increases the cell viability of the 3T3-L1 cells, when the cells are grown in the adipocyte differentiation medium, and may inhibit the differentiation of 3T3-L1 cells from pre-adipocytes into mature adipocytes. Overall, possibly suggesting that Oligo-CS does not promote differentiation of C2C12 cells into adipocytes, and that Oligo-CS inhibits differentiation of C2C12 cells into myotubes through the maintenance of the differentiation state of the cells in myoblasts.

This study demonstrates that oligomerized CS, Oligo-CS, exhibits similar biological effects as high molecular weight CS at low concentrations in the differentiation of RAW264 cells into osteoclasts, and in the differentiation of C2C12 cells into myotubes. Our findings suggest the possibility that Oligo-CS is a promising agent to replace conventional high-molecular weight CS.

## Supporting information

S1 File High resolution images of pictures shown in Fig 2A and 2B. (PDF)

S2 File Raw and analyzed data for the graphs shown in Figs 2C, 3, 4C, 4D, 5, and 6. (PDF)

S3 File Uncropped images for Western blotting data shown in Fig 4B. (PDF)

S4 File High resolution images of pictures shown in Fig 4A. (PDF)
