# Supplementary material for: Effects of chondroitin sulfate oligosaccharides on osteoclast differentiation of RAW264 cells, and myotube differentiation of C2C12 cells
Source: PLoS One. 2023 Apr 13;18(4):e0284343. doi: 10.1371/journal.pone.0284343 (PMC10101473; doi:10.1371/journal.pone.0284343)
Supplement: S1 File — (PDF) [file pone.0284343.s001.pdf]

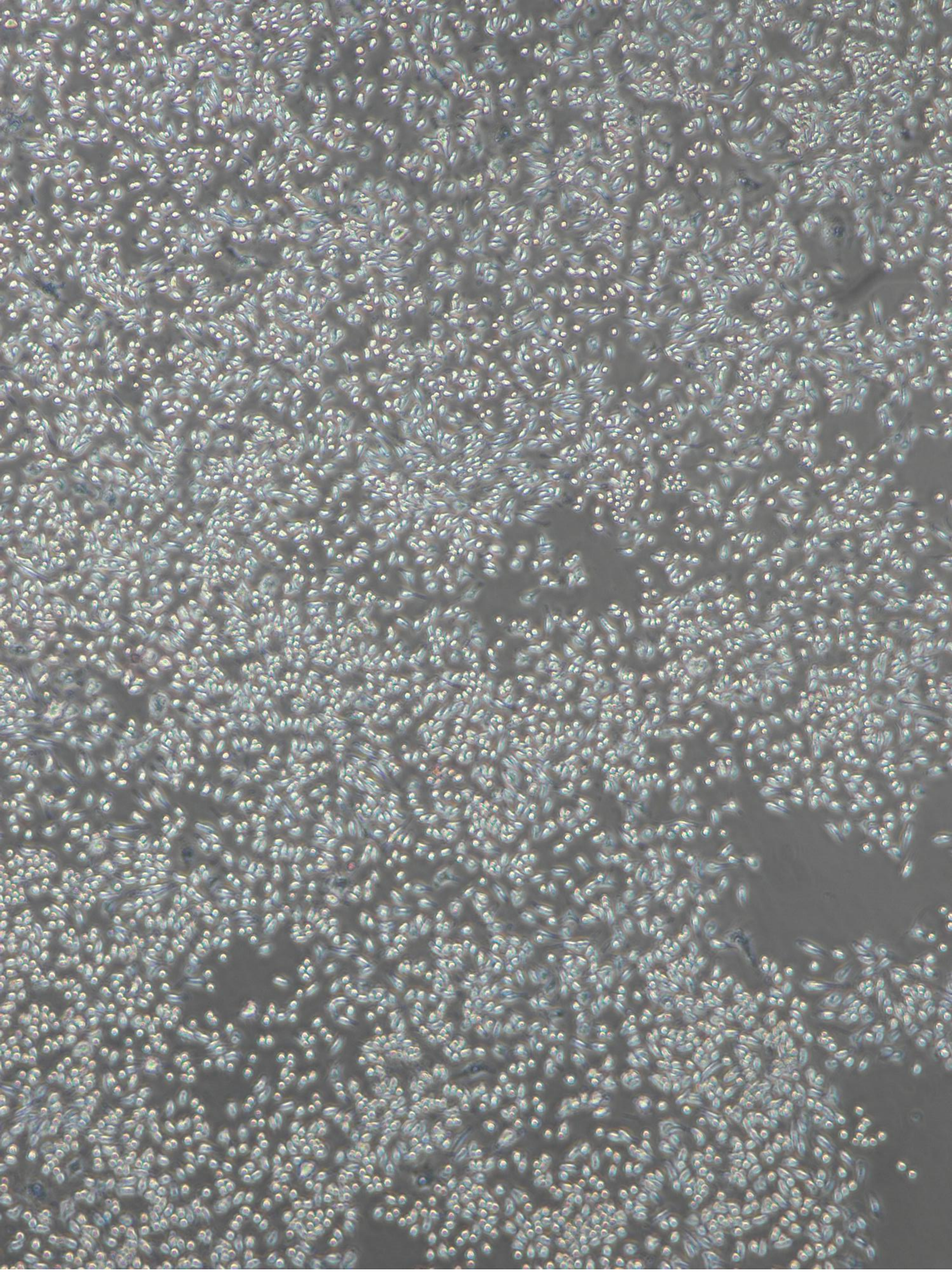

Fig 2A Untreated control cells

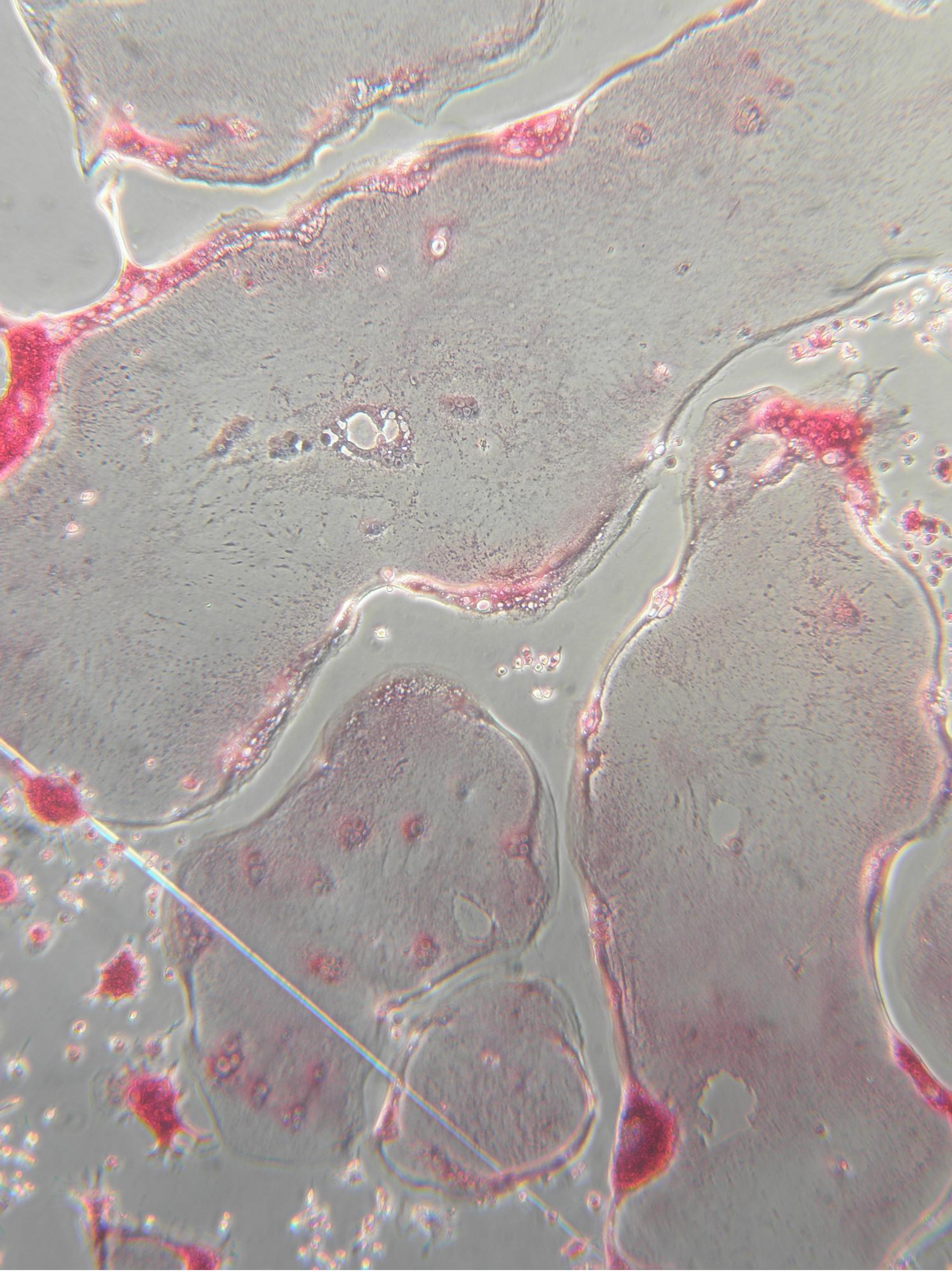

Fig 2A sRANKL treated control cells

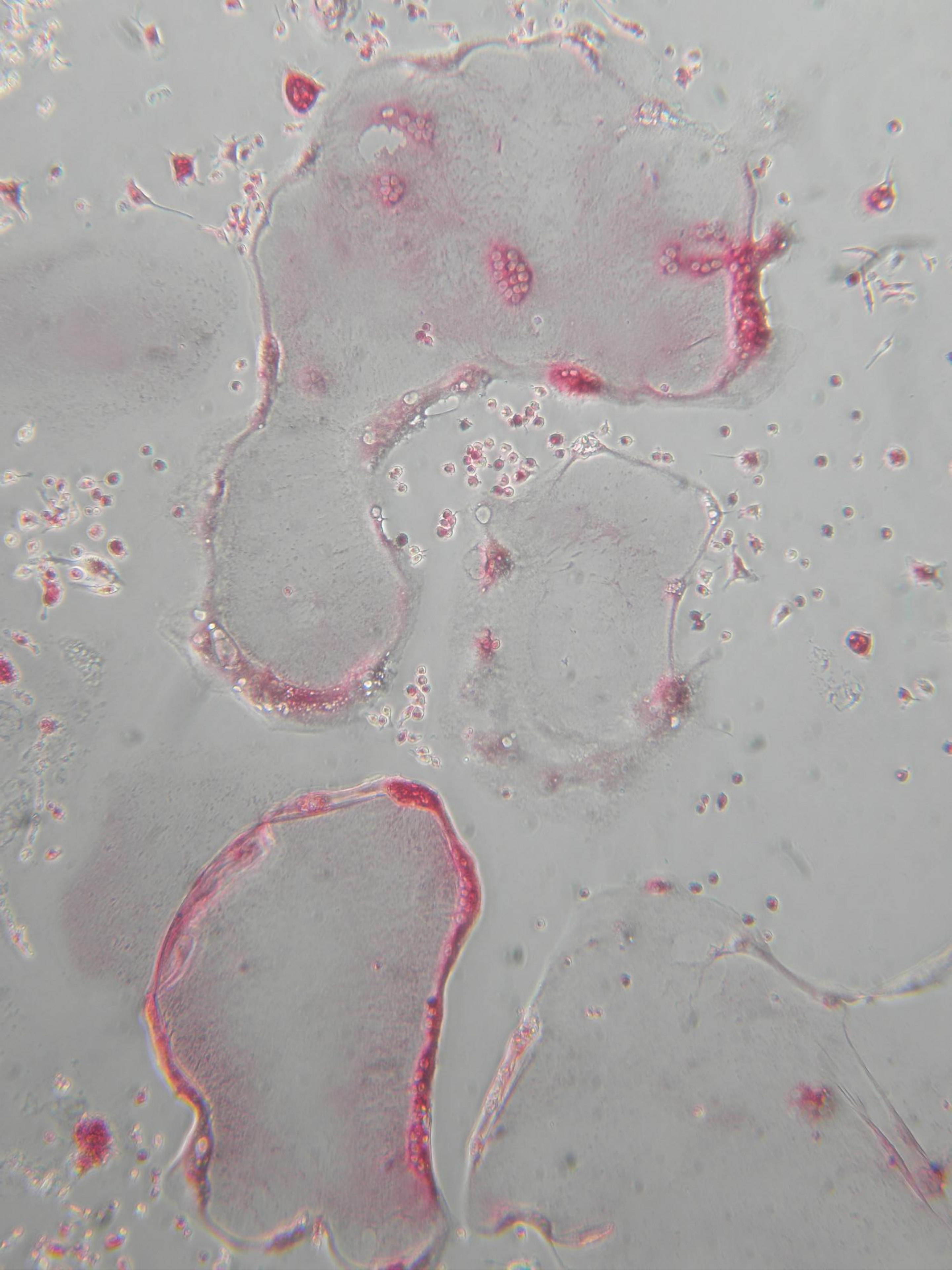

Fig 2B 100 µg/ml CS treated cells

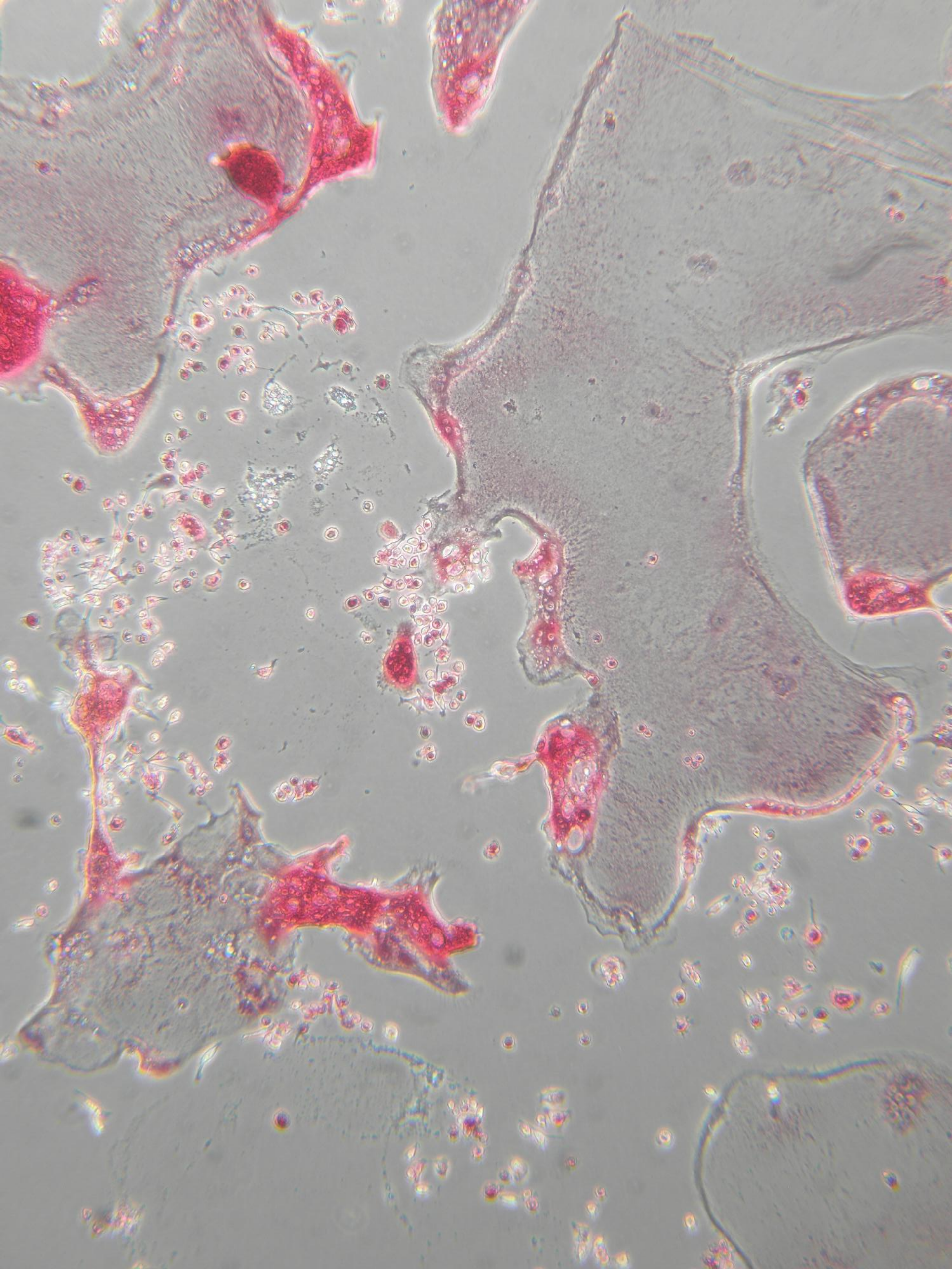

Fig 2B 1000 μg/ml CS treated cells

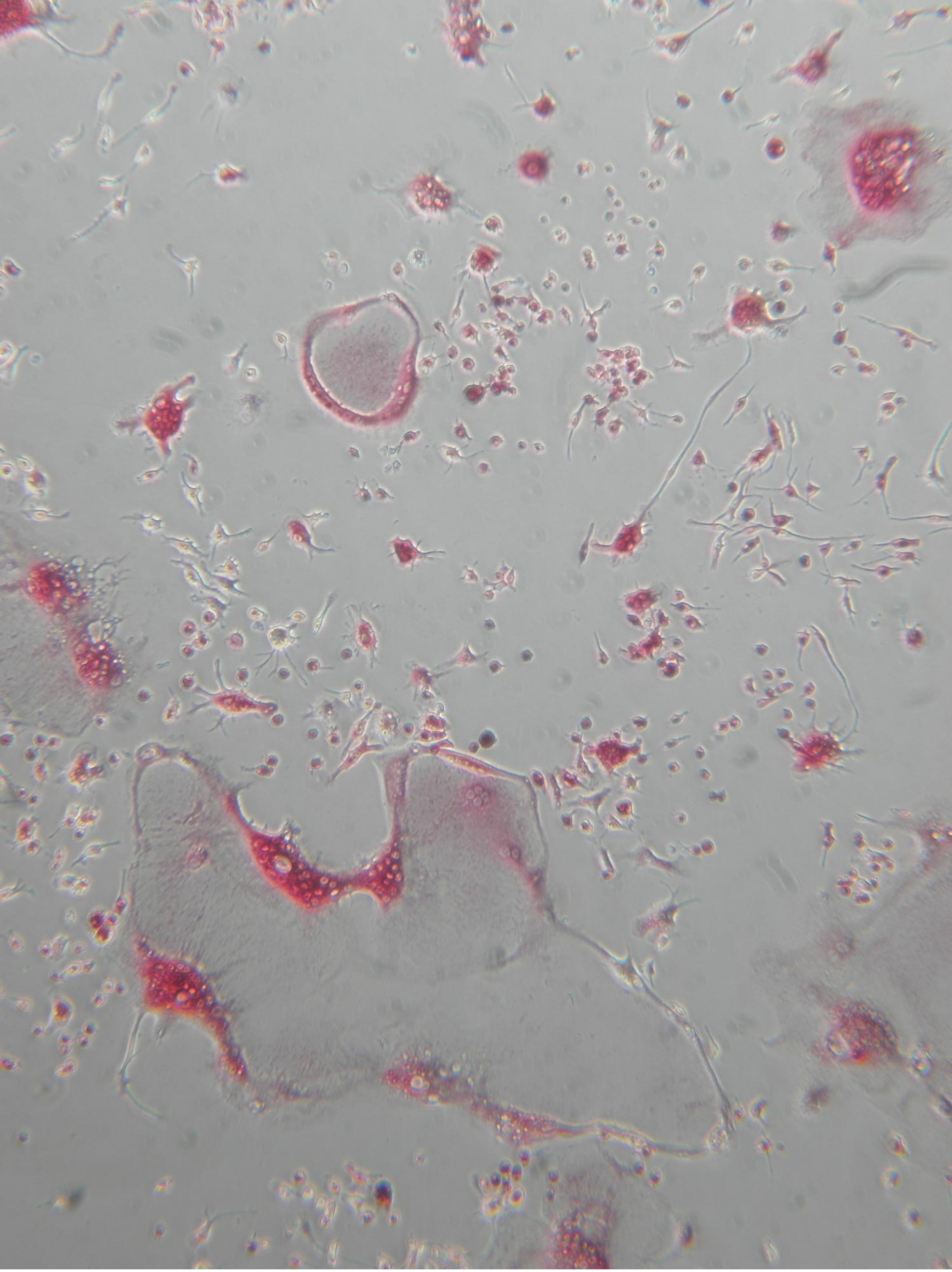

Fig 2B 100 µg/ml Oligo-CS treated cells

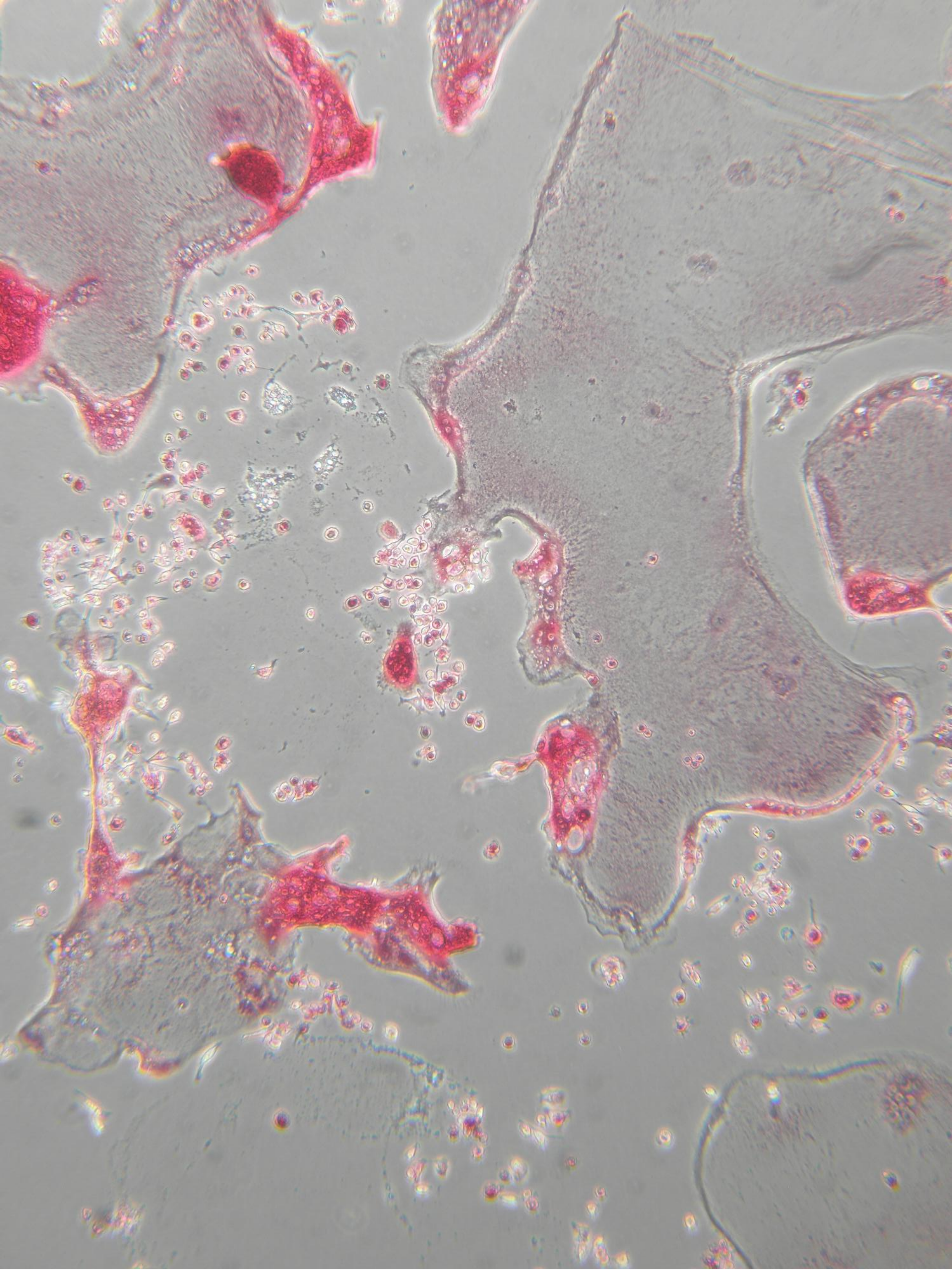

Fig 2B 1000 µg/ml Oligo-CS treated cells
