# Supplementary material for: Effects of chondroitin sulfate oligosaccharides on osteoclast differentiation of RAW264 cells, and myotube differentiation of C2C12 cells
Source: PLoS One. 2023 Apr 13;18(4):e0284343. doi: 10.1371/journal.pone.0284343 (PMC10101473; doi:10.1371/journal.pone.0284343)
Supplement: S2 File — (PDF) [file pone.0284343.s002.pdf]

Analyzed data for Fig 2C

	Average Mature Chondrocyte No. / View			Average	±SD	p-value†
	Experiment 1	Experiment 2	Experiment 3			
Control	5.143	5.429	4.000	4.857	0.756	
CS (100 μg/ml)	5.286	4.333	4.000	4.540	0.667	0.61492
CS (1000 μg/ml)	2.636	2.300	2.571	2.503	0.178	0.02737
Oilgo-CS (100 μg/ml)	3.133	3.000	2.778	2.970	0.180	0.04317
Oligo-CS (1000 μg/ml)	2.778	3.000	2.643	2.807	0.180	0.03638

† The p-values were calculated by comparison to the control.

Raw data for Fig 2C

View	Mature	Immature	View	Mature	Immature	View	Mature	Immature	View	Mature	Immature
**Control**			**CS (100 µg/ml)**			**Oligo-CS (100 µg/ml)**			**Oligo-CS (1000 µg/ml)**		
**Experiment 1**			**Experiment 1**			**Experiment 1**			**Experiment 1**		
1	5	11	1	7	3	1	1	4	1	4	10
2	2	21	2	8	7	2	3	8	2	1	20
3	3	6	3	6	11	3	3	5	3	2	6
4	6	3	4	4	8	4	1	17	4	1	14
5	8	7	5	4	8	5	3	6	5	3	8
6	6	14	6	6	20	6	5	13	6	3	9
7	6	1	7	2	14	7	3	13	7	5	10
**Experiment 2**			**Experiment 2**			8	4	0	8	4	8
8	7	3	8	3	8	9	5	5	9	6	16
9	4	9	9	3	13	10	3	4	10	2	9
10	4	8	10	4	6	11	1	8	11	4	10
11	8	3	11	3	14	12	4	6	12	3	11
12	5	5	12	5	9	13	3	16	13	1	9
13	4	7	13	4	1	14	5	8	**Experiment 2**		
14	6	3	14	5	7	15	3	8	14	3	12
**Experiment 3**			15	6	5	**Experiment 2**			15	2	22
15	4	3	16	6	13	16	5	2	16	2	11
16	6	9	**Experiment 3**			17	1	12	17	2	8
17	2	5	17	3	8	18	5	6	18	1	5
18	3	7	18	2	14	19	2	8	19	0	11
19	5	3	19	3	10	20	2	6	20	4	11
			20	5	2	21	3	7	21	4	14
			21	4	8	22	2	11	22	5	6
			22	2	8	23	3	3	23	1	6
			23	6	5	24	1	11	24	5	3
			24	6	1	25	2	7	25	0	16
			25	5	2	26	2	8	26	3	11
			26	4	4	27	4	8	27	5	8
						28	3	4	**Experiment 3**		
			**CS (1000µg/ml)**			29	4	9	28	2	14
			**Experiment 1**			30	6	3	29	6	17
			1	4	7	**Experiment 3**			30	5	12
			2	1	10	31	3	12	31	5	8
			3	3	2	32	4	6	32	4	10
			4	3	6	33	1	4	33	5	9
			5	2	8	34	2	13	34	3	7
			6	3	12	35	5	11	35	3	10
			7	1	8	36	0	9	36	2	9
			8	3	2	37	3	10	37	4	5
			9	4	6	38	2	8	38	2	8
			10	3	6	39	2	14			
			11	2	8	40	4	4			
			**Experiment 2**			41	5	20			
			12	1	5	42	2	2			
			13	0	18	43	2	8			
			14	7	1	44	3	12			
			15	2	7	45	4	2			
			16	5	5	46	3	4			
			17	2	8	47	2	10			
			18	0	7	48	3	8			
			19	0	8						
			20	2	3						
			21	4	9						
			**Experiment 3**								
			22	4	7						
			23	4	10						
			24	1	10						
			25	2	9						
			26	2	6						
			27	3	10						
			28	2	8						

Raw and analyzed data for Fig 3

		Ct			Fold Change	+SD	-SD	*p*-value[†]
***GAPDH* (Internal Control)**								
	Control	17.18	16.86	16.88				
(+) sRANKL	Control	16.77	17.05	17.06				
(+) sRANKL	CS (100µg/ml)	17.07	17.25	17.13				
(+) sRANKL	CS (1000µg/ml)	17.85	17.60	17.41				
(+) sRANKL	Oligo-CS (100µg/ml)	17.50	17.26	17.29				
(+) sRANKL	Oligo-CS (1000µg/ml)	16.51	16.23	16.25				
TRAP								
	Control	27.04	27.45	27.16	1.00	0.211	0.175	
(+) sRANKL	Control	18.92	19.12	18.80	305.85	53.041	45.202	
(+) sRANKL	CS (100µg/ml)	19.50	19.23	19.59	247.85	38.551	33.362	0.10226
(+) sRANKL	CS (1000µg/ml)	20.33	20.43	20.28	183.12	32.175	27.367	0.02594
(+) sRANKL	Oligo-CS (100µg/ml)	19.99	20.06	19.93	194.01	20.671	18.680	0.03054
(+) sRANKL	Oligo-CS (1000µg/ml)	19.28	19.50	19.45	143.34	20.650	18.050	0.01566
CTSK								
	Control	23.33	23.09	23.50	1.00	0.208	0.189	
(+) sRANKL	Control	19.29	19.07	18.93	18.34	3.394	3.114	
(+) sRANKL	CS (100µg/ml)	19.35	19.35	19.17	18.29	1.844	1.757	0.94904
(+) sRANKL	CS (1000µg/ml)	20.06	19.75	20.51	14.39	5.148	4.401	0.24088
(+) sRANKL	Oligo-CS (100µg/ml)	19.38	19.10	19.45	20.73	3.527	3.257	0.30798
(+) sRANKL	Oligo-CS (1000µg/ml)	19.12	19.62	19.11	10.41	2.683	2.387	0.01078
MMP-9								
	Control	27.94	28.38	27.72	1.00	0.302	0.232	
(+) sRANKL	Control	22.42	22.31	22.63	46.74	8.131	6.926	
(+) sRANKL	CS (100µg/ml)	22.22	22.18	22.31	61.96	5.062	4.679	0.02163
(+) sRANKL	CS (1000µg/ml)	23.18	23.23	23.57	40.32	9.532	7.709	0.22825
(+) sRANKL	Oligo-CS (100µg/ml)	22.56	22.92	22.93	48.06	9.016	7.592	0.79109
(+) sRANKL	Oligo-CS (1000µg/ml)	22.46	22.26	22.31	32.60	4.529	3.977	0.02242

[†] The *p*-values were calculated by comparison to the sRANKL stimulated control.

Raw and analyzed data for Fig 4C and 4D

	Absorbance (450nm)			Cell viability (%)		*p*-value †
	Sample 1	Sample 2	Sample 3	Average	±SD	
**Growth Medium**						
Control	1.1104	1.1338	1.1892	100.00	3.536	
CS (100 μg/ml)	1.0621	1.0570	1.0930	93.55	1.702	0.06861
CS (1000 μg/ml)	1.1017	1.1056	1.1436	97.60	2.023	0.37822
Oligo-CS (100 μg/ml)	1.0934	1.0959	1.1626	97.63	3.430	0.45091
Oligo-CS (1000 μg/ml)	1.1004	1.1432	1.1640	99.25	2.833	0.78884
**Differntiation Medium**						
Control	0.3208	0.2998	0.3757	100.00	11.801	
CS (100 μg/ml)	0.3496	0.3368	0.3868	107.72	7.821	0.40596
CS (1000 μg/ml)	0.3312	0.3360	0.3298	100.07	0.979	0.99272
Oligo-CS (100 μg/ml)	0.3151	0.2865	0.3610	96.62	11.317	0.73824
Oligo-CS (1000 μg/ml)	1.0540	1.0471	1.1534	326.66	17.910	0.00014

† The *p*-values were calculated by comparison to the control.

Raw and analyzed data for Fig 5

	Ct (Target gene)			Fold Change	+SD	-SD	*p*-value[†]
GAPDH (Internal Control)							
Control	16.59	16.25	16.30	1.000	0.358	0.264	
CS (100μg/ml)	16.14	15.92	15.82	0.810	0.196	0.158	0.41729
CS (1000μg/ml)	16.52	16.53	15.93	0.652	0.234	0.172	0.18628
Oligo-CS (100μg/ml)	16.17	16.43	16.35	0.837	0.179	0.147	0.47436
Oligo-CS (1000μg/ml)	16.20	16.25	16.14	0.474	0.041	0.038	0.09459
MyoD							
Control	21.46	21.79	20.99	1.000	0.358	0.264	
CS (100μg/ml)	21.10	21.19	21.60	0.810	0.196	0.158	0.41729
CS (1000μg/ml)	21.68	22.23	22.02	0.652	0.234	0.172	0.18628
Oligo-CS (100μg/ml)	21.45	21.89	21.48	0.837	0.179	0.147	0.47436
Oligo-CS (1000μg/ml)	22.33	22.40	22.19	0.474	0.041	0.038	0.09459
Myf5							
Control	22.44	22.81	22.62	1.000	0.198	0.165	
CS (100μg/ml)	22.32	22.09	22.23	0.993	0.148	0.129	0.96072
CS (1000μg/ml)	22.84	22.82	22.92	0.818	0.223	0.175	0.30393
Oligo-CS (100μg/ml)	22.68	22.62	22.56	0.959	0.102	0.092	0.75309
Oligo-CS (1000μg/ml)	21.27	21.51	21.56	1.991	0.240	0.215	0.00477
Msx2							
Control	27.60	27.41	27.66	1.000	0.169	0.145	
CS (100μg/ml)	26.80	27.21	27.52	0.973	0.308	0.234	0.88749
CS (1000μg/ml)	27.39	27.07	27.53	1.128	0.378	0.283	0.58544
Oligo-CS (100μg/ml)	27.43	27.59	27.74	0.937	0.143	0.124	0.62522
Oligo-CS (1000μg/ml)	26.79	27.08	26.96	1.347	0.154	0.138	0.04818
Sox9							
Control	23.56	23.73	23.41	1.000	0.184	0.155	
CS (100μg/ml)	23.66	23.32	23.58	0.772	0.141	0.119	0.14173
CS (1000μg/ml)	24.43	24.54	24.85	0.469	0.153	0.115	0.01436
Oligo-CS (100μg/ml)	24.36	24.24	24.26	0.581	0.063	0.057	0.03815
Oligo-CS (1000μg/ml)	23.55	23.17	23.49	0.986	0.156	0.135	0.91986
Runx2							
Control	24.05	24.06	24.25	1.000	0.161	0.139	
CS (100μg/ml)	24.20	23.75	24.06	0.810	0.175	0.144	0.20648
CS (1000μg/ml)	25.36	25.05	24.75	0.505	0.189	0.138	0.01755
Oligo-CS (100μg/ml)	24.44	24.27	24.51	0.785	0.105	0.093	0.11612
Oligo-CS (1000μg/ml)	24.45	24.21	24.31	0.765	0.074	0.067	0.09489
PPARγ							
Control	24.20	24.08	24.05	1.000	0.149	0.129	
CS (100μg/ml)	23.56	23.65	23.56	1.072	0.136	0.120	0.54567
CS (1000μg/ml)	23.64	23.24	23.59	1.481	0.483	0.364	0.17558
Oligo-CS (100μg/ml)	23.66	23.58	23.71	1.316	0.143	0.129	0.04747
Oligo-CS (1000μg/ml)	22.69	22.59	22.66	2.428	0.130	0.123	0.00020

[†] The *p*-values were calculated by comparison to the sRANKL stimulated control.

Raw and analyzed data for Fig 6A

	Absorbance (540nm)			Average	±SD	*p*-value
	Sample 1	Sample 2	Sample 3			
Growth Medium	0.438	0.421	0.424	0.428	0.00907	0.00072
Differntiation Medium	0.589	0.562	0.560	0.570	0.01620	

Raw and analyzed data for Fig 6B-G

	Ct			Fold Change	+SD	-SD	*p*-value
***GAPDH* (Internal Control)**							
Growth Medium	17.99	17.83	17.67				
Differentiation Medium	18.29	18.18	18.14				
MyoD							
Growth Medium	24.00	23.65	23.33	1.00	0.294	0.227	0.03668
Differentiation Medium	26.09	26.00	25.80	0.26	0.032	0.029	
Myf5							
Growth Medium	24.19	24.06	24.07	1.00	0.129	0.115	0.00263
Differentiation Medium	22.40	22.19	22.25	4.59	0.444	0.405	
Msx2							
Growth Medium	31.93	32.43	32.22	1.00	0.229	0.186	0.07512
Differentiation Medium	33.22	34.01	32.89	0.57	0.283	0.189	
Sox9							
Growth Medium	26.39	26.17	26.36	1.00	0.148	0.129	0.01932
Differentiation Medium	27.54	27.70	27.51	0.53	0.050	0.046	
Runx2							
Growth Medium	25.29	25.06	25.05	1.00	0.157	0.135	0.21001
Differentiation Medium	25.65	25.69	25.90	0.85	0.096	0.086	
PPARγ							
Growth Medium	24.27	24.10	24.23	1.00	0.135	0.119	0.00061
Differentiation Medium	23.27	23.24	23.13	2.57	0.198	0.184	
