# Supplementary figures and images for: Effects of chondroitin sulfate oligosaccharides on osteoclast differentiation of RAW264 cells, and myotube differentiation of C2C12 cells
Source: PLoS One. 2023 Apr 13;18(4):e0284343. doi: 10.1371/journal.pone.0284343 (PMC10101473; doi:10.1371/journal.pone.0284343)

Anti-Myosin Heavy Chain (MYH)

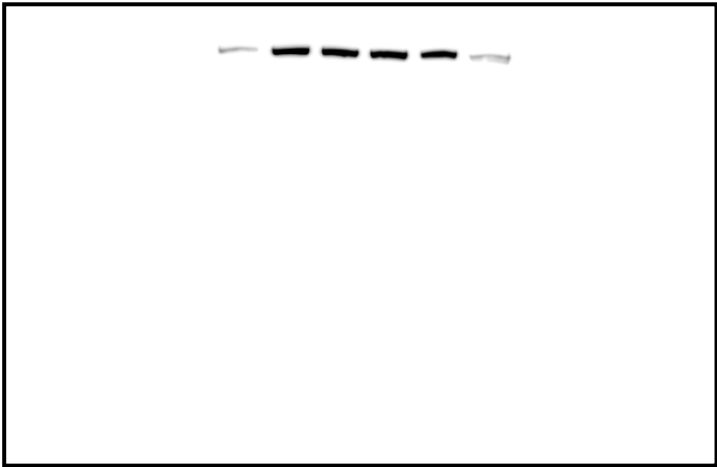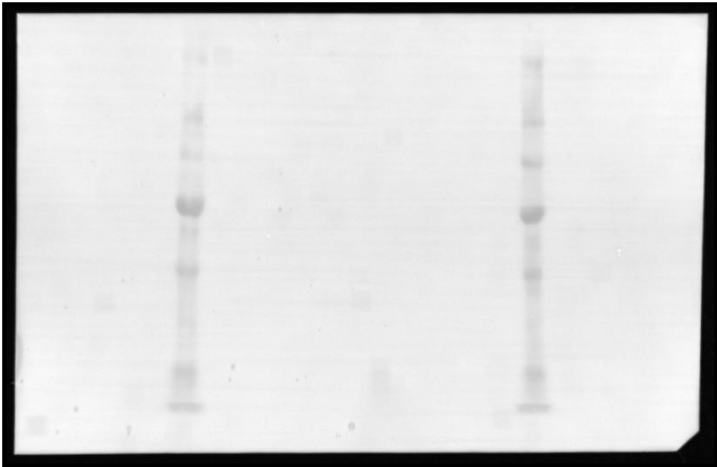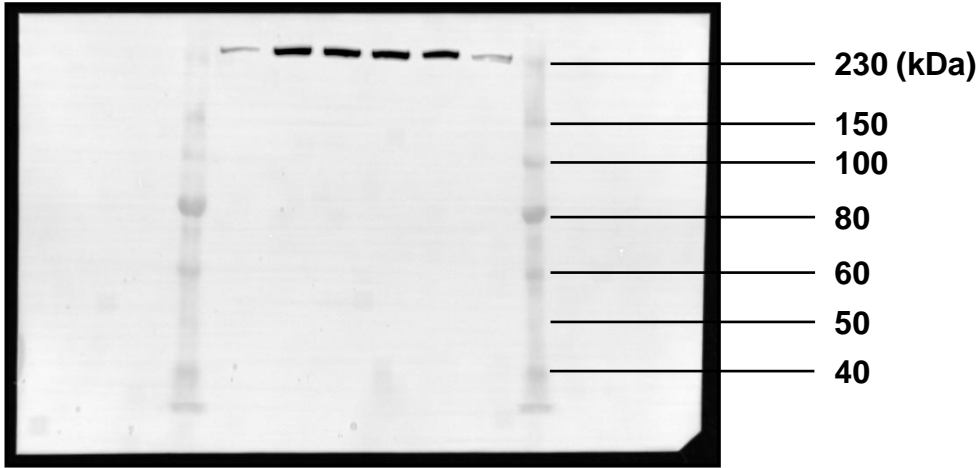

Anti-β-Actin (Reprobing)

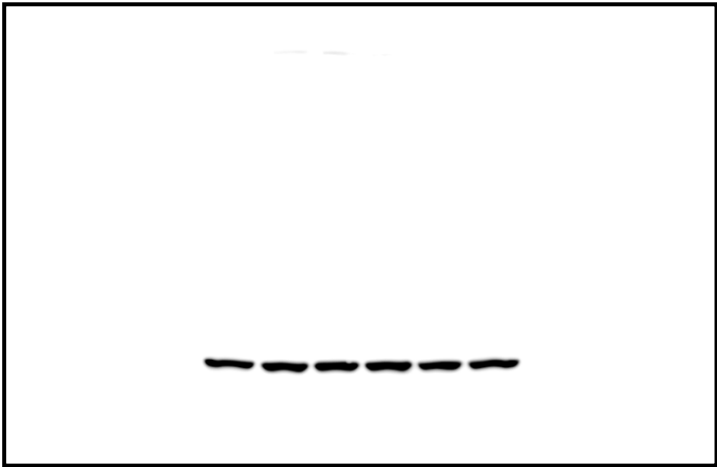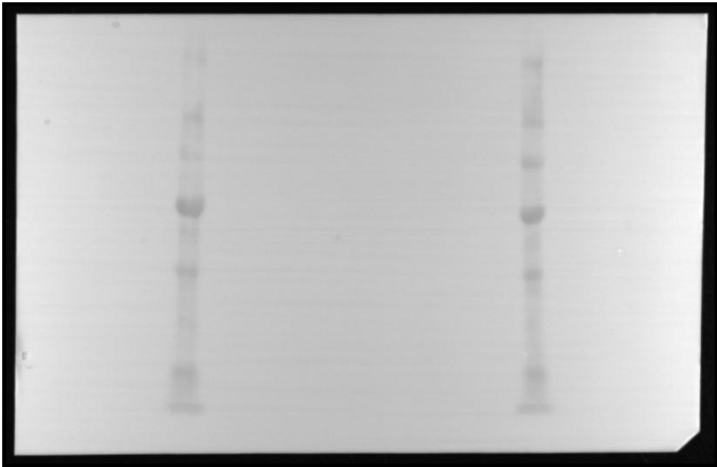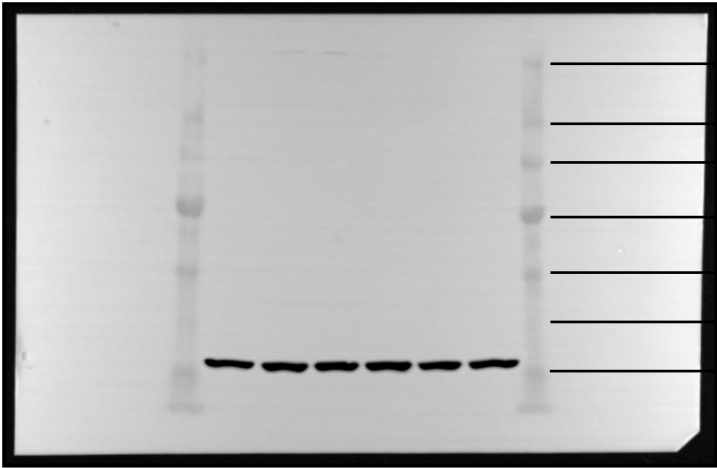

230 (kDa)  
150  
100  
80  
60  
50  
40

Supplement: S3 File — (PDF) [file pone.0284343.s003.pdf]
